# Best paper and the best reviewer of IJU

**DOI:** 10.4103/0970-1591.60434

**Published:** 2010

**Authors:** Nitin S. Kekre

**Affiliations:** Department of Urology, Christian Medical College, Vellore - 632 004, Tamilnadu, India. E-mail: editor@indianjurol.com

The 43^rd^ annual meeting of USI was a great success and the organizing committee of USICON 2010 deserves all the praise for conducting a wonderful meeting in the historical city of Agra. It was also eventful for the Indian Journal of Urology. For the first time we organized an independent evening function to celebrate the journal's achievements. It also provided an opportunity to thank all the people who have participated and helped us in our endeavor. The IJU best paper first prize award was bestowed to Dr. S. Sriram for his paper titled “Detection and Management of post transplant renal artery stenosis” at the hands of USI President Dr. Ganesh Gopalakrishnan. The second best paper award was awarded to Dr. (Mrs) P. Rekha for her paper “Histological reclassification, histochemical characterization and c-kit immunoexpression in renal cell carcinoma” by Dr. S.V. Kotwal, the President Elect.

The editorial board took this opportunity to acknowledge the contribution of all peer reviewers and Professor Robert Pickard, Freeman Hospital, Newcastle, UK was selected as the best international peer reviewer. While Dr. Gagan Gautam was considered as the best reviewer from India for the year 2009. I congratulate both of them and hope that they would continue to help the Indian Journal of Urology.

This issue carries a symposium on “Testicular cancer” edited by Dr. Hemant Tondgonkar, the Head of Urology Department, Tata Cancer Hospital, Mumbai. Testicular cancer is a remarkable success story and a fine example of multidisciplinary management of malignancy.

I would like to take this opportunity to personally express gratitude to the outgoing chairman of the Indian Journal of Urology Dr. Mahesh Desai. He has been a source of great strength and was always available in times of crisis. It was his dream to make the Indian Journal of Urology one of the best and to make it visible in the international arena. I hope that he will continue to guide us in future.

The editorial board welcomes Professor Dr. Ganesh Gopalakrishnan as the new chairman of the Indian Journal of Urology and we all look forward to working with him.

With best wishes.

